# The road less travelled? Exploring the nuanced evolutionary consequences of duplicated genes

**DOI:** 10.1042/EBC20220213

**Published:** 2022-12-08

**Authors:** Emily Anna Baker, Alison Woollard

**Affiliations:** Biochemistry Department, University of Oxford, South Parks Road, Oxford OX1 3QU, U.K.

**Keywords:** Evolutionary biology, Gene duplication, Genetic redundancy

## Abstract

Duplicated genes have long been appreciated as both substrates and catalysts of evolutionary processes. From even the simplest cell to complex multicellular animals and plants, duplicated genes have made immeasurable contributions to the phenotypic evolution of all life on Earth. Not merely drivers of morphological innovation and speciation events, however, gene duplications sculpt the evolution of genetic architecture in ways we are only just coming to understand now we have the experimental tools to do so. As such, the present article revisits our understanding of the ways in which duplicated genes evolve, examining closely the various fates they can adopt in light of recent work that yields insights from studies of paralogues from across the tree of life that challenge the classical framework.

## Introduction

Traditionally, there exists the notion that following their formation, duplicated genes can evolve in one of three ways. First, the adoption of new functionality (via changes to either the regulatory or coding sequence or both) by neofunctionalisation. Second, the degeneration of one of the paralogues resulting in its non-functionality by the process of pseudogenisation. And third, the partial, though necessarily complementary, degeneration of both paralogues such that the expression of the single ancestral gene becomes divided between the new duplicates in a process known as subfunctionalisation.

These three modes of duplicated gene evolution have provided the classical framework for the empirical assessment of paralogue diversification for some 20 years despite not originating from empirical studies. However, it is arguable that no framework (empirically derived or otherwise) should remain unchallenged for such a long length of scientific time given the emergence of new evidence that is hard to fit within it. In any case, it is certainly worth exploring whether ‘pigeonholing’ paralogues into the classical framework inevitably lead to an underestimation of their complexity.

It goes without saying that our current ability to study the structure and function of genes has eclipsed that of previous decades, providing insights into the complexity of genetic architecture that were unknowable when the classical models of paralogue evolution were being developed. And so, in view of such emerging complexities, it is now worth scrutinising our understanding of duplicated gene evolution with the aim of moving towards a more nuanced understanding of the evolutionary consequences of duplicated genes.

## Origin of an idea: duplicated genes as substrates and catalysts

Even before the structure of genes had been characterised, Ohno posited the evolutionary potential of gene duplications in his seminal work ‘*Evolution by Gene Duplication’* [1]. In his book, Ohno emphasised that evolution by natural selection was an inherently conservative process and that only with new genetic material – generated by gene duplication events – could novelty emerge. Although, even prior to Ohno’s work, Lewis had tentatively put forward the idea that new genes arising from duplication, or ‘higher repetition’ could go on to perform *similar* or new functions relative to the pre-existing gene from which they were derived [2]. Though erroneously termed ‘pseudoalleles’ by Lewis (adapting the term from McClintock’s original coinage of it [3]), he still hit upon the same idea that would, decades later, prove to be so transformative and conceptually critical to the fields of evolutionary biology and genetics as we know them today.

Beyond its establishment as a revolutionary idea, the concept of gene duplications as a powerful force in evolution remained mechanistically vague until the late 1990s when it was outlined exactly how they might actually catalyse complex evolutionary processes [4,5]. With the aim of addressing a paradox in Ohno’s thinking, pioneers of the field Michael Lynch and Allan Force sought to explain why so many duplicated genes persist over the course of evolution (not undergoing pseudogenisation) if neofunctionalisation only occurs in the rarest of circumstances. Their answer came in the form of the Duplication-Degeneration-Complementation (DDC) model, known more commonly as subfunctionalisation. Force and Lynch rooted this third mode of paralogue evolution in regulatory divergence as *the* means of functional differentiation and in so doing constructed the framework, for studies of duplicated gene evolution for the 20 years that followed.

At the same time as ideas about their evolutionary fates were being developed, the molecular origins of duplicated genes also started to become apparent [6–8]. While it is beyond the scope of the present article to describe them in detail, the mechanisms by which genes are duplicated require outlining if only in brief for our purposes.

Gene duplication events can broadly be categorised in two ways: small-scale and whole-genome duplication (WGD) events. Taking the first, and most common gene duplication mechanism, small-scale events include tandem duplications which may arise in a variety of ways, namely – non-allelic homologous recombination associated with unequal exchange between sister chromatids [9]; unequal crossing over in meiosis prophase I [10]; or non-homologous recombination repairing chromosomal breakages caused by replication going awry [11]. Small-scale duplication events of this sort are mediated by repetitive elements, such as tandem or inverted repeats, which inadvertently provides regions of homology thereby facilitating recombination. Therefore, depending on the point of recombination, and by extension the location of the repeat, part of a gene, a whole gene or several genes can be duplicated at once.

Small-scale duplications, however, do not necessarily yield paralogues in tandem. Transposition, or more commonly retrotransposition, also have the potential to duplicate entire genes. Retrotransposition occurs when a messenger RNA (mRNA) is retrotranscribed into complementary DNA (cDNA) and subsequently inserted into the genome. The hallmarks of retrotransposed duplicates are traditionally considered to be a lack of introns and the absence of regulatory sequences such as a promoter. It is for the latter reason that paralogues generated via retrotransposition were long thought to merely pseudogenise shortly after their genesis [12]. However, it has been reported that up to 12.5% of all human retrogenes – of which there are ∼8000 – are in fact transcribed [13,14], suggesting that some may escape their fate by co-opting the regulatory apparatus of neighbouring genes or by generating their own *de novo*. In contrast, other studies suggest that as little as 4% of human retrogenes are everexpressed [15]. Despite the lack of consensus regarding their transcriptional potential, it is known that paralogues derived from retrotransposition can create introns from internal exonic sequences, a phenomenon known as ‘intronisation’ [16]. For this reason, the presence of introns alone cannot be taken as definitive evidence to reject retrotransposition as the mechanism of paralogue generation in any given instance. Thus, it may be the case that the number of functional paralogues derived from retrotransposition has been historically underestimated.

In contrast with its small-scale counterpart, the mechanism of WGD remains elusive. What is known is that there are two sorts, autopolyploidisation and allopolyploidisation. The first involves a WGD event within a species, which may feasibly occur if cytokinesis fails during early development, or if prior to this at fertilisation there is pronuclear fusion involving an unreduced gamete [17]. The latter might arise if there is a failure in meiosis II, or an inability to extrude the polar bodies at the end of meiosis. The second – allopolyploidisation – is the mutational product of an interspecies cross, which almost without exception is thought to lead to sterility due to inevitable mitotic failures when attempting to pair similar, but crucially non-homologous, chromosomes [17].

The distinction between the possible mechanisms of WGD is not trivial. This is because the main advantage to deriving paralogues through WGD as opposed to small-scale events is considered to be the maintenance of stoichiometry – all subunits of protein complexes, genes and their regulators, and so on, are simultaneously duplicated such that there are no subsequent dosage imbalances [1,18,19]. This is heralded as WGD’s major advantage over tandem duplication and explains why paralogues derived from WGD persist over longer periods of evolutionary time compared with any other sort [19–21]. In other words, what these events wreak in mutational consequence they make up for in stoichiometric integrity [1]. However, this is surely only the case for paralogues derived from autopolyploidisation as those that originate from allopolyploidisation – depending on the extent of the divergence between the two species crossing to generate the initial polyploid – are not necessarily able to interact faithfully. Of course, this would wholly depend on the interaction in question as some interactions (e.g., certain transcription factor (TF)-binding preferences) are inherently promiscuous such that any sequence differences between new paralogues may in fact be inconsequential or could even generate novel targets.

## Beyond a mere paradigm of paralogues

In this article, the *nuanced* consequences of duplicated genes refer to the exploration of their fates in light of their origins and genetic context. In particular, we review how the coevolution of paralogues is constrained by their unduplicated regulatory elements, and the fates they are free, or not free, to assume in light of their molecular origin. With this in mind, let us first consider the fates duplicated genes have been shown to adopt which appear to fall outside the classical tripartite paradigm.

### Alternative fates of duplicated genes

The conventional wisdom regarding the fates of duplicated genes was bound to be too simple. Indeed, no author has ever claimed that those are the *only* foreseeable ways in which duplicated genes can evolve. And nor could they, because for as long as the classical models have existed, it has been appreciated that paralogues can maintain genetic redundancy with one another for considerably long periods of evolutionary time [22]. This is a state of affairs that should not be permitted in the classical view because one paralogue would inevitably be under relaxed selection making true redundancy evolutionarily unstable and thus transient [5]. On the contrary, redundancy is thought to be a pervasive phenomenon among paralogues generally [22], but this lacks any explanation in the classical framework.

One such explanation, though, may be found in an alternative fate known as hypofunctionalisation [23]. Hypofunctionalisation involves the reduction in the expression of *both* duplicated genes such that both must be retained by necessity and so are unable to diverge over time [20,23,24]. However, if paralogues acquire the ability to compensate for one another (e.g., by up-regulation of expression), then neither paralogue would have a phenotype if it were to be mutated – redundancy would therefore ensue. This is not subfunctionalisation in the classical sense because functionality (in terms of expression domains) has not been lost, that is to say paralogue expression is not spatially distinct. A recently characterised example illuminates hypofunctionalisation dynamics.

T-box TFs are an uncharacteristically large and dynamic family in the *Caenorhabditis* genus of nematodes. Among two of the 21 paralogues in *Caenorhabditis elegans* are *tbx-37* and *tbx-38*, known for their redundant role in mesodermal induction in the early embryo [25]. Originally put forward as a case of true redundancy, recent work suggests that all are not what it first seems when it comes to the interaction between these two paralogues. When endogenous reporter constructs of *tbx-37* and *tbx-38* were made, each was only observable in the early embryo when the other paralogue was knocked out implying each has the capacity to be up-regulated when its counterpart is compromised [26]. And so, it would seem that *tbx-37* and *tbx-38* are paralogues locked into a hypofunctionalised relationship – ordinarily (i.e., in the wild-type scenario) expressed at low levels individually that combine to produce the full expression potential required for normal development. Presumably, this combined expression recapitulates the level of expression associated with the ancestral single-copy orthologue, and the regulatory interaction between *tbx-37* and *tbx-38* has evolved as a compensation mechanism if one paralogue was to deteriorate. It is hard to conceive of another reason for maintaining duplicated genes in such a state as this other than for the purpose of instilling robustness in a gene regulatory network. Indeed, this may be of paramount importance for paralogues involved in critical aspects of development or if they belong to rapidly evolving, mutationally vulnerable, gene families (both statements that apply to *tbx-37* and *tbx-38*). Thus, hypofunctionalisation falls out with the classical framework for the fates of duplicated genes and adequately explains genetic redundancy in some cases but is unlikely to always explain instances of persistent redundancy between paralogues.

Another explanation for the maintenance of redundant duplicated genes over long periods of evolutionary time may lie in pleiotropy, i.e., the ability of certain genes to perform multiple functions. The redundancy between *erd-2.1* and *erd-2.2* in *C. elegans* is reminiscent of many such relationships between other paralogous KDEL receptors in the animal kingdom [22,27]. Simultaneous knockdown of *erd-2.1* and *erd-2.2* is lethal, yet when abolished individually there are no obvious phenotypic consequences. While not characterised as such in *C. elegans*, work in budding yeast [28] and *Drosophila* [29] suggests the role of the ERD family is to retrieve proteins that have been trafficked to the Golgi apparatus. The Erd paralogues in *C. elegans* are 84% similar in amino acid sequence, and exactly why they have remained as such for approximately 80 million years of *Caenorhabditis* evolution remained mysterious until it became known that they are both able to moonlight as (equally capable) suppressors of deleterious mutations in the acetylcholine transporter, UNC-17 [27]. It was deduced that while certain mutations compromised the primary function of the Erd paralogues as receptors, those same mutations, surprisingly, made them able to suppress certain deleterious UNC-17 alleles. And while this kind of allele-specific suppression may at first seem highly coincidental and artefactual, it may on closer inspection lend a suitable explanation for the persistence of an additional Erd paralogue as a genetic ‘spare tyre’.

In permitting survival of otherwise deleterious mutants, the preservation of both *erd-2.1* and *erd-2.2* as almost identical genes actually creates their functional potential as regulators of neurotransmission. So more than simply instilling robustness in their basic role as cellular traffickers, maintaining redundant Erd paralogues permits both paralogues the opportunity to flexibly adopt a new function, which would not be accessible to a single-copy orthologue, nor to two divergent Erd paralogues where the roles of the receptor and suppressor were permanently divided between two different genes. In this way, these kinds of long-standing redundancies may, paradoxically, open up striking adaptive opportunities. This differs from neofunctionalisation and subfunctionalisation in that latent pleiotropy maintains the redundancy over evolutionary timescales. But what about moonlighting more broadly? Surely, gene duplication offers the perfect solution to single-copy genes that are required to multitask in seemingly unrelated biological scenarios.

Unlike the latent pleiotropy exhibited by the Erd paralogues, some gene products in the natural world display moonlighting behaviour (which can be defined as the ability of a single-gene product to perform two or more biochemically unrelated, independent functions [30]). In such a scenario, it might seem as though subfunctionalisation (though necessarily with respect to coding sequence) would be the obvious route for newly duplicated moonlighters to take. However, this only rarely occurs [31]. Stoichiometric limitations may be relevant (among others) if the functions of the gene product are associated with the same biological context, such as response to a particular environmental perturbation. Such a limitation might have led to the fates of the partially redundant pair of paralogous moonlighters Hxk1 and Hxk2 in budding yeast [32,33]. These two genes catalyse the phosphorylation of hexoses but both moonlight as transcriptional regulators of Glk1 required for the metabolism of aldohexoses [33]. Hxk1 and Hxk2 are expressed under different nutritional conditions yet can fully compensate for the loss of their paralogue by up-regulating their expression irrespective of the nutrients present. Their compensatory dynamic is not dissimilar to the interaction between *tbx-37* and *tbx-38* in *C. elegans*, only more drastically so, because Hxk1 and Hxk2 are not ordinarily coexpressed at all. This is to say that Hxk1 and Hxk2 are *specialised* for moonlighting under different circumstances.

Specialisation is more accurately defined as a form of asymmetric paralogue divergence where one paralogue becomes adept at a distinct aspect of the ancestral gene’s function, possibly even elaborating on it, while the other retains a broad association with the ancestor [34,35]. Recent studies have found specialisation to be a common phenomenon in large multigene families, the *C. elegans* Warthogs being one such gene family.

The Warthogs are a family of ten genes with partial homology to the Hedgehog ligand belonging to the signal transduction pathway of the same name [34,36]. Exclusively found in the nematode phylum, the Warthogs are products of many tandem gene duplication events. Ancestrally involved in ecdysis, the extant crop of Warthogs fall into two classes: the first being ‘generalists’ that exhibit a range of low penetrance, additive, moulting phenotypes; the second being ‘specialists’ that display redundancy in their role synthesising the cuticle anew. The generalists instil robustness in moulting by signalling in different pathways, while the smaller number of specialists impart robustness in the process of cuticle biogenesis through their classical redundancy. In any event, the loss of ancestral functionality and their specialisation in either shedding the old cuticle or synthesising the new has permitted the Warthogs to assume novel roles in at least four aspects of post-embryonic development [34,37]. And so, it may be hypothesised more generally that specialisation enables members of gene families to adopt a divide and conquer strategy of functional *innovation* not available to sub-functionalised duplicates.

### Regulatory coevolution among coevolving paralogues

A conceptual flaw in the field-defining notion that newly duplicated genes (derived from small-scale events) have an untapped evolutionary potential is that genes are not merely their promoters, introns, and exons. For a gene to give rise to its phenotype, it is reliant on regulatory elements often located far away from its coding region. Thus, it is axiomatic that new, initially identical, duplicates are not free to assume any imaginable fate given they are inevitably limited by demands on their unduplicated regulatory elements. In the next section, we shall explore the ways in which paralogues resolve such regulatory conflicts to delineate their expression from one another and how these shape the fates of the duplicates in question.

Two recent illustrations of how this regulatory crossroads can be resolved come from studies in *Drosophila melanogaster*. The first to provide insights into this is the *bric-a-brac* (*bab*) locus, comprising the tandemly duplicated genes *bab1* and *bab2* that share a single enhancer [38]. While the enhancer regulates overlapping *bab1* and *bab2* expression along the proximodistal axis of the developing leg disc, *bab2*-specific expression in the leg disc was also shown to be under the control of the same enhancer; but how? It was postulated that divergence in the *bab1* and *bab2* promoters facilitates unique interactions with the TF Rotund at the *bab2* locus. The shared enhancer can respond to Rotund, affecting both *bab1* and *bab2* expression, but it is thought the additional interaction with the *bab2* promoter is responsible for the subsequent recruitment of additional repressors that directly act on *bab2* to delimit its expression [39,40].

Second, and mechanistically redolent of the action of *bab1* and *bab2* in the leg disc, is the expression of the functionally redundant *pdm2* and *nub* paralogues in the developing wing disc [41]. Again, the expression of both paralogues relies on a shared enhancer, but their ability to respond to it differs – *nub* responds in all wing progenitor cells and *pdm2* only in a small subset. This differential response is a result of a *pdm2*-specific silencer element in the *pdm2* promoter that receives repressive input from Rotund. And crucially, the repression by Rotund depends on *nub*, allowing *pdm2* to fully respond to the wing enhancer when *nub* expression is compromised thereby enabling functional compensation to occur.

Elaboration on pre-existing regulatory programmes is the most well-trodden route (that we know of) to refining the expression of duplicated genes. However, as the cases of *bab1*/*bab2* and *pdm2*/*nub* demonstrate, their origins by tandem duplication result in both paralogues being linked and under the command of the same *cis*-regulatory element, which is clearly restrictive with respect to the ability of paralogues to functionally diverge from one another. This is just one reason why the fates of paralogous genes are ultimately constrained by the particular duplication event from which they were derived.

### Context-dependence and the limitations on duplicated genes

It seems intuitive that both the mechanism and age of duplication limit the ability of paralogues to persist over evolutionary time because it makes them more or less prone to pseudogenisation (by being more or less able to become functionally distinct). It was not, however, until relatively recently that these ideas were supported by empirical evidence from studies of plant genome evolution [42]. By analysing the genomes of 25 taxonomically diverse plant species, the ability of duplicated genes to be retained over the course of evolution in light of their duplication mechanism, gene function, and age of duplication were all investigated [43]. It was found that tandem paralogues tend to be much younger compared with those that originate from WGD events, but that regardless of how they are derived, TFs are overwhelmingly retained following their duplication. An explanation for why this might be could lie in the behaviour of TFs compared with other genes. The highly interactive nature of TFs means that their stoichiometry is critical [43,44]. Thus, it could be argued that TFs should either be lost almost immediately following their duplication or forced to rapidly assume a new function to avoid such a loss.

A hypothesis for why it is that TFs are disproportionately retained following their duplication relates to expression attenuation [45]. This is thought to protect against the particularly harmful short-term consequences of duplicating TFs, which act as the major regulators of organismal development. However, it may be that following their down-regulation, paralogous TFs are required to evolve much slower as they hypofunctionalise and become unable to diversify – effectively trapped into working as one gene because the stakes are simply too high to subsequently gain or lose functions [21,43–45]. This may explain why redundancy is so prevalent among duplicated TFs especially, and flies in the face of the narrative that suggests acquiring new transcriptional regulators is always a path to new evolutionary opportunities.

## Conclusion

*“I shall be telling this with a sigh*

*Somewhere ages and ages hence:*

*Two roads diverged in a wood, and I—*

*I took the one less traveled by*,

*And that has made all the difference.”*

*‘The Road Not Taken’ by Robert Frost* [46]

**Figure 1:**
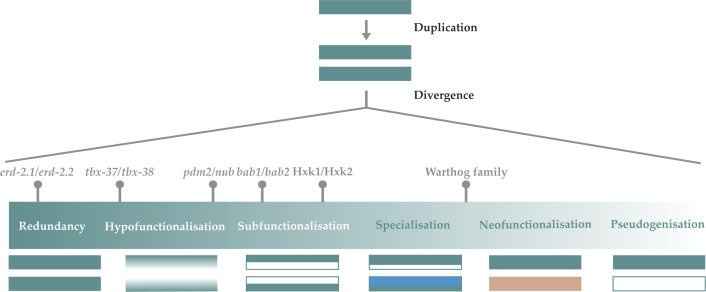
Endless fates most bountiful The spectrum of possible duplicated gene fates is depicted according to the functional similarity between paralogues, where complete functional redundancy (far left) represents that the greatest degree of functional similarity that can be exhibited and pseudogenisation (far right) the least. Genes are represented by rectangles with their colour denoting their function (teal depicting the ancestral gene function; white depicting the loss of ancestral function; sky blue depicting an elaborated function as in the case of specialisation; and pink depicting new functionality as in the case of neofunctionalisation). Throughout the middle of the spectrum there exist a multitude of potential paralogous gene fates with all degrees of functional overlap possible between them. The experimentally determined fates of paralogues discussed in the present article (*tbx-37/38*; *erd-2.1/erd-2.2*; Hxk1/Hxk2; the Warthog family; *bab1/bab2*; and *pdm2/nub*) are mapped onto the spectrum.

There are endless ways for duplicated genes to evolve; indeed, as many ways as there are paralogous genes. Figure 1 describes the fates of duplicated genes as falling on a spectrum, which while including the classical fates, elaborates and extends them in light of recent empirical observations. It is impossible to give any more than a flavour of all the evolutionary fates of duplicated genes in one article, but this is the point. Our ability to study genetic complexity is now enabling us to catch a glimpse of the staggering array of paralogue dynamics and tease apart the many nuances of the fates duplicated genes adopt. While themes emerge from such studies, these fates should not be thought of as strict evolutionary identities that duplicated genes assume in discrete classes. No two sets of paralogues share the same context, be this their unique combinations of age, origin, regulatory demands, and interacting partners. And so, it follows that no two sets of paralogues could ever be expected to share the same fate. It is with this in mind that we as evolutionary geneticists would do well to remember the words of the American poet Robert Frost. Rather than restricting our understanding of paralogue evolution to the three arterial fates, it is incumbent on us to embrace, and seek to understand, the idiosyncratic paths trodden by duplicated genes to meet their own adaptive ends.

## Summary

- Our understanding of how duplicated genes evolve has been shaped by a framework – based on theoretical considerations – in which duplicated gene fates are categorised in one of three ways: neofunctionalisation, pseudogenisation, and subfunctionalisation.
- This classical framework has defined the scope and interpretation of empirical assessments of paralogue evolution for two decades such that the theory has not only lagged behind the insights derived from empirical studies, but has actually limited them.
- In the present article, we discuss examples of paralogue dynamics which fall outside the classical framework, suggesting there are in fact many more ways that duplicated genes can evolve than just three.
- We argue that due to the unique combinations of paralogue age, mechanistic origin, regulatory constraints, and interacting partners (among others), no paralogues could ever be said to adopt a canonical evolutionary fate – this is to say that there are as many fates as there are paralogous genes.
- To this end, it is incumbent on us to seek to understand and embrace the idiosyncratic paths trodden by duplicated genes, and in so doing achieve a more nuanced understanding of the roles they play in the evolution of development.

## Open Access

Open access for the present article was enabled by the participation of University of Oxford in an all-inclusive *Read & Publish* agreement with Portland Press and the Biochemical Society under a transformative agreement with JISC.

## Abbreviations

cDNA: complementary DNA
DDC: duplication-degeneration-complementation
TF: transcription factor
WGD: whole-genome duplication
